# Microstructured Lipid Carriers (MLC) Based on N-Acetylcysteine and Chitosan Preventing *Pseudomonas aeruginosa* Biofilm

**DOI:** 10.3390/ijms22020891

**Published:** 2021-01-17

**Authors:** Marta Guerini, Pietro Grisoli, Cristina Pane, Paola Perugini

**Affiliations:** Department of Drug Sciences, University of Pavia, Via Taramelli 12, 27100 Pavia, Italy; marta.guerini01@universitadipavia.it (M.G.); pietro.grisoli@unipv.it (P.G.); cristina.pane01@universitadipavia.it (C.P.)

**Keywords:** microstructured lipid carriers, N-acetylcysteine, chitosan, biofilm, *Pseudomonas aeruginosa*, antioxidant activity, cystic fibrosis

## Abstract

The aim of this work was the development of microstructured lipid carriers (MLC) based on chitosan (CH) and containing N-acetylcysteine (NAC), a mucolytic and antioxidant agent, to inhibit the formation of *Pseudomonas aeruginosa* biofilm. MLC were prepared using the high shear homogenization technique. The MLC were characterized for morphology, particle size, Z potential, encapsulation efficiency and drug release. The antioxidant properties of NAC-loaded microstructured carriers were evaluated through an in vitro spectrophotometer assay. Finally, the activity of NAC-CH-MLC on biofilm production by *Pseudomonas aeruginosa* was also evaluated. Results obtained from this study highlighted that the use of chitosan into the inner aqueous phase permitted to obtain microstructured particles with a narrow size range and with good encapsulation efficiency. NAC-loaded MLC showed higher antioxidant activity than the free molecule, demonstrating how encapsulation increases the antioxidant effect of the molecule. Furthermore, the reduction of biofilm growth resulted extremely high with MLC being 64.74% ± 6.2% and 83.74% ± 9.95%, respectively, at 0.5 mg/mL and 2 mg/mL. In conclusion, this work represents a favorable technological strategy against diseases in which bacterial biofilm is relevant, such as cystic fibrosis.

## 1. Introduction

Cystic fibrosis (CF) is a genetic disorder caused by mutations of a gene encoding a multifunctional protein, the cystic fibrosis transmembrane regulator (CFTR), expressed on the apical membrane of epithelial cells and submucosal glands [1].

CTFR represents a 1480 residue-long membrane protein, component of the ATP-binding cassette (ABC) transporter family and it functions as an ion channel; the presence of a supplementary regulatory region and N- and C-terminal extensions constituted by 80 and 30 residues in length, respectively, are a distinctive feature of this protein [2]. The epithelial disfunction of CFTR causes an imbalance between chloride and epithelial sodium channels (ENaC)-mediated sodium absorption, causing dehydration of the airway surface liquid and thickening of the mucus [1]. The loss of ion conductance across the membrane of these cells produces impaired ion and liquid homeostasis, causing a multiorgan disorder [3]; the organs mainly affected are the lungs, pancreas, liver, gut and the reproductive system (especially in males, due to the obstruction of the spermatic ducts) [2].

Among rare diseases, CF is the most frequent, and it is a cause of early mortality in Caucasians worldwide. This autosomal recessive inherited disease occurs in approximately 1 in 3000–4000 live births. In the US, approximately one in 25–30 Caucasians are carriers of a pathogenic mutation of the CFTR gene; in other ethnicities, CF occurs less commonly, including approximately 1 in 4000–10,000 Latin Americans, 1 in 15,000–20,000 African Americans, and even less commonly in Asian Americans. Outside the US, CF registries exist in Canada, the United Kingdom, Europe and Oceania, all of which are used to better identify the number of individuals with CF and the course of the disease [4].

The major cause of death in CF patients consists of progressive pulmonary disease; many in vitro studies indicate that CTFR dysfunction may directly alter airway immunity through increased production of proinflammatory mediators and impaired immune response to pathogens [5]. The thickness of CF mucus is due to the presence of huge amounts of DNA, actin, proteases and pro-oxidative enzymes originating from a subset of inflammatory cells (neutrophils). The hyperinflammatory syndrome of CF lungs has several bases: there is an imbalance between proinflammatory and anti-inflammatory cytokines, especially IL-8 and IL-10, respectively [6]; chronic oxidative stress linked to the release of oxidants by neutrophils and to abnormal antioxidant defense, caused by malabsorption of dietary antioxidants, and a defect in glutathione (GSH) efflux [7].This hyperinflammatory syndrome associated with pH lowering caused by the damage of CTFR protein could predispose chronic infections sustained by opportunistic bacterial pathogens. The most common bacterium which infects CF lungs is *Pseudomonas aeruginosa* mucoid phenotype, a gram-negative microorganism. This bacterium forms a biofilm inside the lung, which prevents the penetration and therefore the action of antibiotics. Furthermore, the presence of extracellular DNA, elastase and radical products facilitates the expression of the mucoid phenotype of *Pseudomonas* [8,9], as the excessive presence of neutrophils around the biofilm leads to the mutation of a gene, *mucA*, which causes the appearance of the mucoid phenotype [10].

CTFR-related dysregulation of respiratory epithelial cells and innate immune dysfunction in the lung determines disproportionate airway inflammation, which hinders efficient clearance of the pulmonary pathogen [5]. Similarly, periodontal infections are mainly caused by pathogens organized in biofilm, such as *Porphyromonas gingivalis* or *Aggregatibacter actinomycetemcomitans*; periodontitis results in endothelial damage and periodontal tissue destruction due to the host inflammatory response achieved through oxidative stress pathways [11,12]. Therefore, in these diseases, it is necessary to counteract the presence of pathogenic microorganisms; in particular, a redox therapy may be useful to prevent biofilm formation in CF.

N-acetylcysteine (also known as N-acetyl cysteine, N-acetyl-L-cysteine or NAC) is a tripeptide and acetylated precursor of the amino acid L-cysteine: it has been considered a mucolytic agent [13] since 1960, thanks to its ability to disrupt disulfide bridges in the high-molecular-weight glycoprotein of mucus. The antioxidant activity of NAC, closely linked to GSH level in the blood, was widely recognized in 1980; NAC is also known to be the antidote for paracetamol intoxication, it is mostly administered orally, but it can be administered even by inhalation or intravenously [14].

The relative low toxicity and mild side effects, like nausea, vomiting, rhinorrhea and pruritus, make this drug safe and widely used in the many clinical fields [14,15].

The first pharmacokinetic parameter related to oral administration of NAC is attributed to the work of Borgstrom et al. [16]; the author proved that the bioavailability varies with formulation: it is lowered by the oral route with individual values ranging between 3.4% and 18.8%. Looking at the oral bioavailability of NAC, the drug can be considered in two ways: the reduced form, as the parent drug, and its metabolites or all the NAC, regardless of their redox status. Olsson et al. [17] assessed the oral pharmacokinetic: his work showed the achievement of a maximum concentration of NAC in a reduced form of 3.47 mg/L after 30 min, translated into a bioavailability of 4% or 9.1% considering total NAC (t ½: 6.25 h).

The administration of NAC intravenously has been used for several therapeutic indications and mainly for the treatment of liver detoxification due to paracetamol poisoning, but few studies are available about NAC concentrations in plasma and its pharmacokinetics when it is administered by intravenous route.

The inhalation of NAC is generally limited to pulmonary therapy, in which the drug efficiently exerts its direct action as a mucolytic agent; in fact, as previously mentioned, inhaled NAC as well as other thiol derivates act mainly on the lower respiratory tract, making the mucus thinner, as the main target of these drug is the mucin [18]. However, this administration route has some disadvantages, for example local irritation, cough and bronchospasm, due to the high acidity of the compound (pH 2.2). Moreover, the unpleasant smell of sulfur typical of NAC has to be considered when inhaled. Some studies show that inhaled NAC is effective on oxidative stress, advising that patients with higher oxidative stress may benefit from inhaled NAC therapy, as the glutathione replenished by NAC inhalation can reverse oxidant–antioxidant imbalance [19,20]. Homma et al. in 2012 with a multicenter, prospective, randomized controlled trial showed that monotherapy with inhaled NAC had beneficial effects in patients with idiopathic pulmonary fibrosis (IPF) [21], differently from the PANTHER trial which demonstrated that the monotherapy with NAC administered orally had no effect in IPF patients [22].

Miller A.C. et al. in 2009 [23] undertook a single-center, retrospective, controlled study: the author used aerosolized unfractionated heparin combinate with N-acetyl-L-cysteine, and the results underlined attenuation of lung injury and the progression of acute respiratory distress syndrome in ventilated adult patients with acute lung injury following smoke inhalation; thus, the mechanism of action of NAC inhalation is still disputed and further studies assessing different delivery methods of NAC are required.

In light of this, NAC can therefore be considered for drug repurposing, or drug re-profiling, that is, attributing a new pharmacological use to an existing drug, presenting some advantages, such as a chemical aspect already known, as well as toxicity, safety and pharmacokinetic profile [24,25].

The main aim of this study was the development of NAC-loaded microstructured lipid carriers (MLC) for pulmonary application. Microstructured lipid carriers, characterized by a blend of solid lipid and oil, were chosen to overcome the lipids β-modification during storage, and consequently drug expulsion from the lipid matrix [26]. Lipid microparticles with small diameter and very narrow size distribution were chosen in the present work, since these specific dimensions can appear more suitable for drug delivery in the respiratory tract and for their higher stability during nasal aerosol administration.

Usually, the payload of hydrophilic compounds into lipid nanoparticles is very low. During the last years, several strategies have been employed to avoid this problem, like the development of oil-loaded nanoparticles, (nanostructured lipid carriers, NLC) or the modification of the lipid matrix by incorporation of thickening or amphiphilic agents [27].

In the present work, chitosan (CH) was used as an active excipient in order to increase NAC stability and to prolong drug residence time in the action site. Chitosan [(1 + 4)-2-amino-2-deoxy-p-Dglucan] is a hydrophilic cationic polymer obtained by N-deacetylation of chitin, with low toxicity (oral LD50 in rats is 16 g kg^−1^). Furthermore, its biodegradability and biocompatibility were finely established [28]. Mucoadhesion is one of the several properties that make this polymer a useful pharmaceutical excipient; Leher et al. [29] suggest that the mucoadhesive properties of CH are probably a result of ionic interaction between the positively charged amino groups in CH and the negatively charged sialic acid residues in mucus. This polymer was successfully employed in prolonging drug residence time in the eye by administration of acyclovir-loaded chitosan microspheres [30]. Furthermore, chitosan was already used as conjugate with NAC to increase its molecular weight and to promote active permeation through membranes [31].

CH was employed in this work into the inner phase and not for the coating, as reported in literature. This choice was made in order to maintain the hydrophilic active into the MLC and to prevent its diffusion to the outer hydrophilic phase during microparticle formation. In addition, CH can promote a modified release of NAC and exert a mucoadhesive effect when the matrix degrades, adhering to the mucous membrane of the lung.

Considering these purposes, the physicochemical characterization of NAC-loaded MLCs, the evaluation of their in vitro antioxidant effectiveness, the drug release profile and their capability to prevent *P. aeruginosa* biofilm formation were studied.

## 2. Results

### 2.1. Microparticle Preparation and Characterization

**Figure 1:**
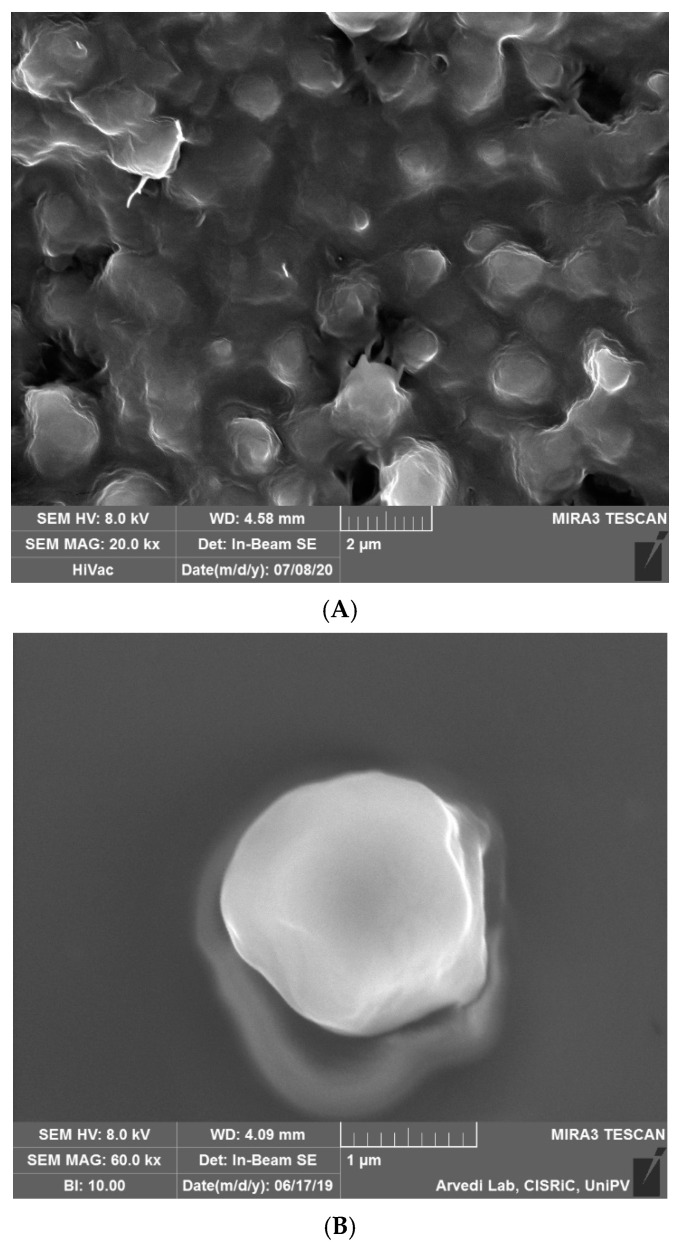
SEM images of (**A**) microstructured lipid carriers (NAC-CH-MLC); (**B**) single particle at higher magnification.

A high shear homogenization technique was used in the present work in order to obtain solid lipid particles. SEM analyses (Figure 1) investigated the shape and the dimension of the solid carriers. The shape appeared regular and spherical, while the diameter seemed to be around 1 μm. The MLC surface appeared smooth and no drug crystals were evident on the surface.

#### 2.1.1. Dimensions and Zeta Potential

**Figure 2:**
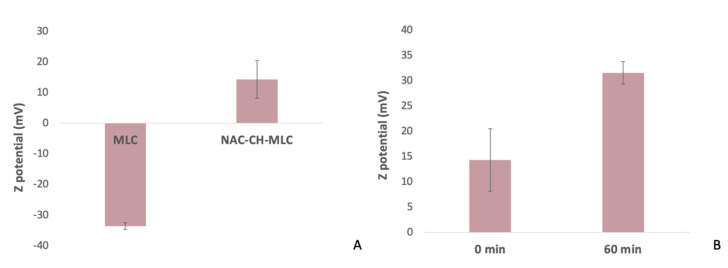
(**A**) MLC and NAC-CH-MLC Z potential; (**B**) NAC-CH-MLC Z potential at 0 min and 60 min of soaking.

The analysis of the dimensions of microparticles shows how MLCs without the addition of CH had smaller dimensions (841.28 nm ± 47.39, P.I. = 0.335) than NAC-CH-MLCs (1023.2 nm ± 71.07, P.I. = 0.351). NAC-CH-MLC dimensions also reflect the examined dimensions of the CH-MLC in Figure 2 (P.I. = 0.12).

**Figure 3:**
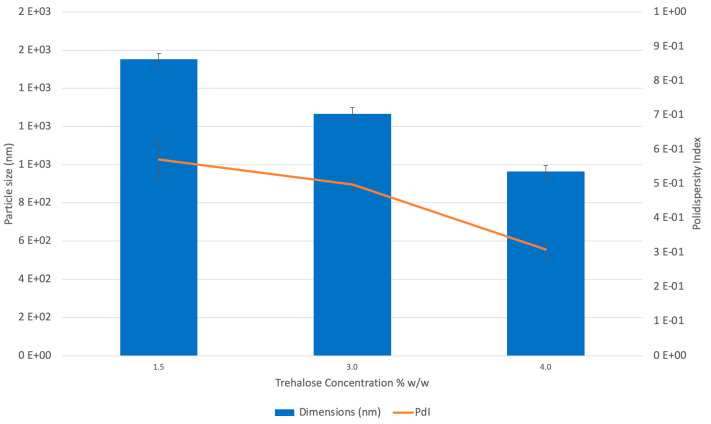
CH-MLC dimensions (nm) and polidispersity index (PdI) at different trehalose concentrations: 1.5% *w*/*w* (batch CH-MLC 1), 3% *w*/*w* (batch CH-MLC 2) and 4% *w*/*w* (batch CH-MLC 3).

Figure 3 shows the relationships between microparticle size or polydispersity index (PdI) and the cryoprotectant concentration. In both cases, with increasing trehalose concentration, the best results concerning the reduction of particle dimensions to 965 ± 30.98 and a PdI equal to 0.313 ± 0.027 (*p* value < 0.05) were obtained.

Figure 2A shows results concerning the zeta potential of MLC; the surface of the MLC is negative (−33.6 ± 1.1 mV), due to the presence of polygliceryl-4 sorbitan olivate phosphate and of glyceryl behenate, which is characterized by a fraction of 12–18% mono-, 52–54% di-, and 28–32% triglycerides; the fatty acid fraction consists of greater than 87% of behenic acid. Freitas C. et al. measured the Z potential of SLN made by 10% of Compritol 888 ATO and 1.2% of poloxamer 188, the result right after production was −24 mV [32]. NAC-CH-MLC, on the other hand, had positive charge of about 14.3 ± 6.16 mV, indicating that the particles effectively acquired a cationic surface due to the presence of CH.

**Figure 4:**
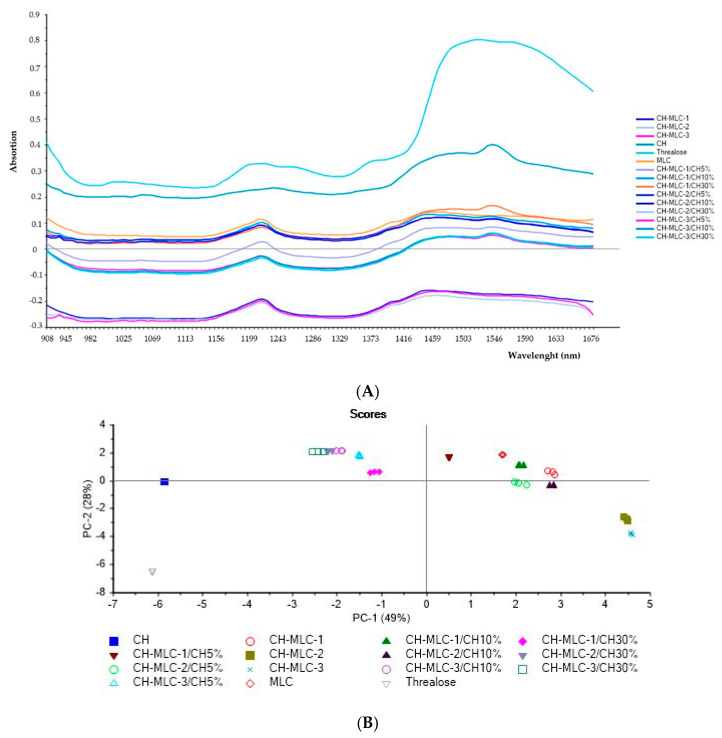

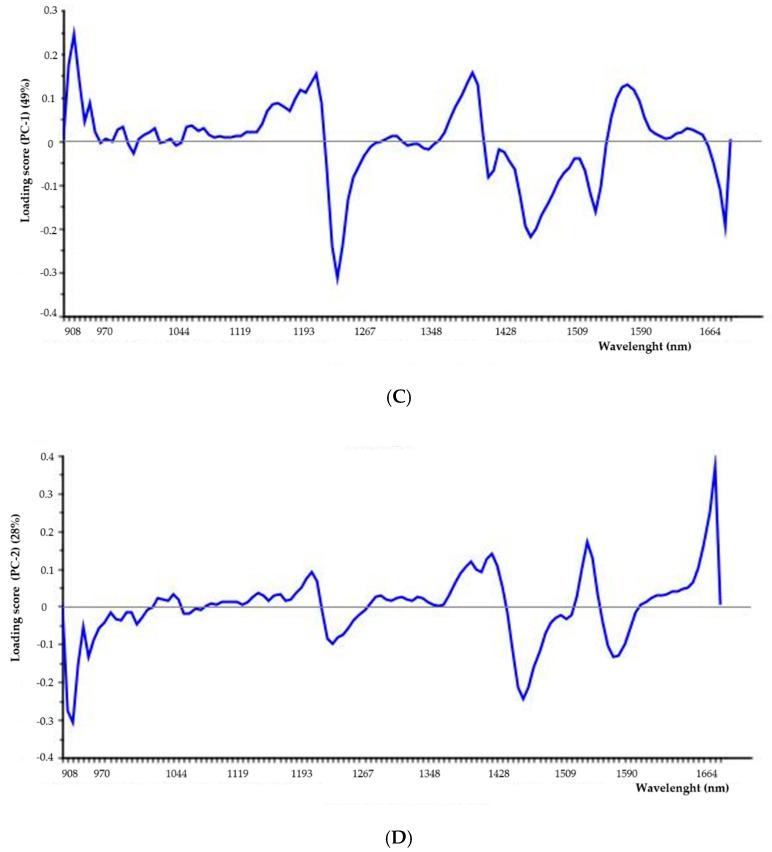
Results from near-infrared (NIR) analysis of chitosan (CH), trehalose powder, placebo microstructured carrier without CH (MLC); placebo microstructured carriers with chitosan (CH-MLC) and physical mixtures between CH and placebo freeze-dried CH-MLC. (**A**) Spectra; (**B**) principal components analysis (PCA) scores plot; (**C**) and (**D**) loadings plots of PC1 and PC2, respectively.

Then, Z potential was measured (Figure 4B) after 60 min of soaking and the obtained value was 31.54 ± 2.23 mV (*p* < 0.05). This behavior underlines the swelling of chitosan with consequent higher exposure of its amino groups to the aqueous solution.

#### 2.1.2. Near-Infrared (NIR) Analysis

The NIR (near-infrared) spectroscopy technique was used for a further nondestructive characterization of MLC in order to reveal the presence of trehalose and CH in microstructured carriers.

All samples were analyzed in triplicate by MicroNIR and data were evaluated using principle component analysis (PCA) on pretreated spectra, as previously explained (Figure 4).

Firstly, as observed from Figure 4A, this technique demonstrated the ability to distinguish the different samples.

In the PCA scores plot (Figure 4B), it is evident that CH and trehalose were distinct and well separated especially along PC-2. The loading plots reported in Figure 4C,D highlighted the principal bands responsible for this separation: the area around 1680 cm^−1^ is typical of the presence of carbonyl of nonsubstituted amide; a band around 1590 cm^−1^, indicates the prevalence of NH_2_ groups. The 1381 cm^−1^ band corresponds to deformation modes with participation of the OH, CH, and C–N groups; in the 1280–1150 cm^−1^ interval antisymmetric and symmetric C−O bond stretching appears [33,34].

Principal components analysis carried out on microstructured carriers revealed that batches containing 3% and 4% w/w of trehalose (CH-MLC-2 and CH-MLC3) were aligned to the trehalose along PC2 demonstrating the presence of the sugar in the MLC freeze-dried samples (Figure 4B).

Moreover, physical mixtures of microparticles:chitosan with higher CH concentration (CH-MLC-1/CH30%; CH-MLC-2/CH30%; CH-MLC-3/CH30%) were aligned to CH along PC2 but differentiated from it along PC-1. These results confirmed the capability of the NIR technique to detect specifically the presence of CH in a complex mixture. Furthermore, comparing the scores of CH-MLC-1 and placebo MLC produced without CH addition, it is clear that they were very close to each other, demonstrating that in the CH-MLC-1 CH amount was too low to be revealed by NIR analysis. On the other hand, CH-MLC-2 and CH-MLC-3, batches produced with the same CH concentration but with higher amount of trehalose, appeared in another area of the PCA plot (Figure 4B).

Finally, batch CH-MLC-3 seemed have the higher amount of CH entrapped. In fact, its physical mixture with only 5% w/w of CH powder permitted to move its score in the PCA plot in the area of physical mixture containing 30% of CH.

### 2.2. HPLC Method Optimization and Validation

Sana S. and al [35] compared, as a mobile phase, buffer:acetonitrile (80:20) and buffer:methanol (80:20), and the use of acetonitrile caused a faster elution of the component. Jyothi N.N. et al. [36] proposed a mobile phase containing phosphate buffer (pH 4.5) and methanol in the ratio of 900:100 (*v*/*v*) to have a better resolution. After several experiments, a symmetric and sharp peak was obtained using parameters reported in Table 1. Specificity was demonstrated by the absence of interference signals around the NAC peak. The NAC peak appears well resolved and distinct from the signal of mobile phase.

**Figure 5:**
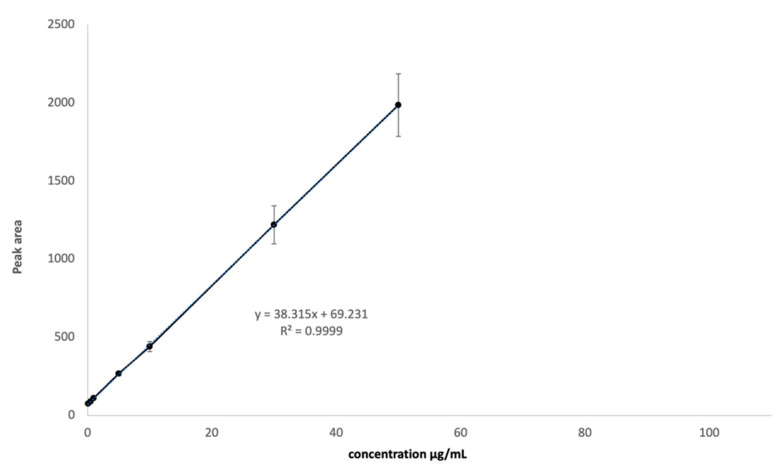
NAC calibration curve in the range between 0.1 and 50 µg/mL.

Linearity was assessed from the calibration plot between concentration and peak area, and the correlation coefficient of the curve was above 0.9999, as shown in Table 2. Accuracy was demonstrated as mean % recovery and it was found to be 100.69%, with values ranging from 99.69% to 101.25%. Method precision showed a %RSD of 1.53%. LOD and LOQ values were, respectively, 0.54 and 1.65 µg/mL. Then, a calibration curve was built and used for the following determination of the amount of NAC recovered in MLC (Figure 5).

### 2.3. N-Acetylcysteine Release

**Figure 6:**
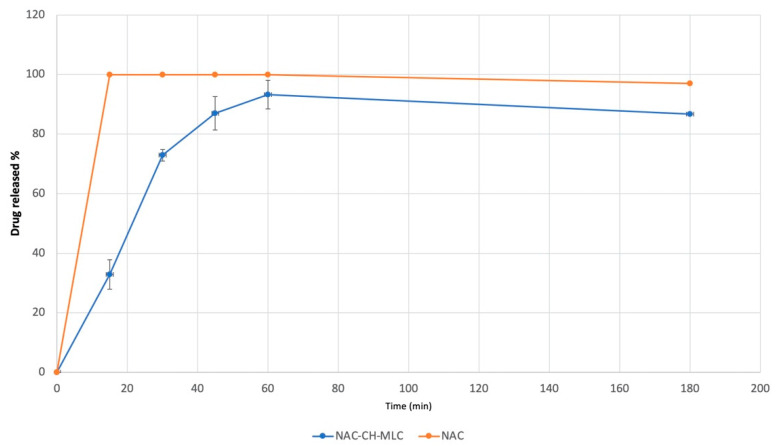
N-acetylcysteine release profile from NAC-CH-MLC and NAC dissolution profile in NaCl 0.9%.

Figure 6 depicts the release profiles of N-acetylcysteine from lyophilized MLC, dispersed in a NaCl 0.9% *w/v* solution, and the dissolution profile of NAC powder as control. NAC is a very hydrophilic compound, thus its dissolution is very quick, reaching 100% of solubilized drug at 15 min. MLC demonstrated to be able to modulate drug release, showing at 15 min a NAC release of 32.91% ± 4.91%, reaching the maximum amount of NAC released after 60 min (93.25% ± 4.79%). Every test was repeated three times and results are expressed by mean value of the triplicates. Moreover, standard deviation and coefficient of variation were calculated for all samples.

### 2.4. Occlusive Properties of MLC

**Figure 7:**
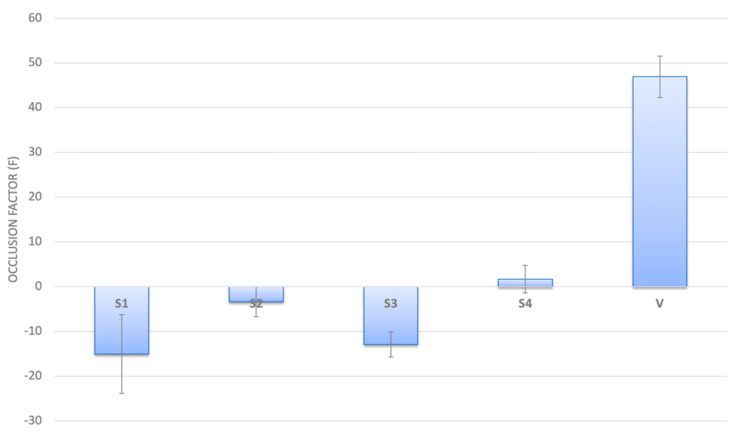
Occlusion Factor (F) after 24 h from application of S1 (NAC-CH-MLC at concentration of 12 mg/cm^2^), S2 (NAC-CH-MLC at concentration of 50 mg/cm^2^); S3 and S4 are CH-MLC samples with the same solid concentration of S1 and S2, respectively; V is the occlusive standard.

Occlusive properties of MLC were performed using two different solid concentrations in order to predict a probable negative effect in following experiments with microbial population. Results reported in Figure 7 highlighted that neither NAC-CH-MLC nor CH-MLC had any occlusive properties.

### 2.5. Antioxidant Activity

DPPH is a free radical capable of reacting and shrinking to form hydrazine DPPHH. The antioxidant compounds, in fact, are able to transfer to the radical a hydrogen atom, causing the discoloration of the solution and a peak reduction at 516 nm [37].

The kinetics of reaction between NAC and DPPH showed that the plateau was reached after 5 min (data not reported), thus NAC can be classified under the fast kinetic antioxidant category. Ascorbic acid is used as a control standard for this category [38].

Preliminary analyses were made to demonstrate that the use of a mixture of acetonitrile and lactic acid, useful for MLC dissolution, did not interfere with the measurement of the antioxidant activity of the drug.

The EC50 value calculated for NAC in its free form was 0.23 ± 0.02 mg/mL. The obtained value was then compared with the EC50 of NAC inside MLC (0.15 ± 0.01 mg/mL) (Table 1). For simplicity, these values have been converted to their reciprocal, the higher the ARP, the greater the ability of the active compound to reduce the radical. EC50 and the corresponding ARP values for ascorbic acid were reported from literature being about 0.17 and 5.9, respectively [38], very similar to the NAC value obtained from the present study.

**Figure 8:**
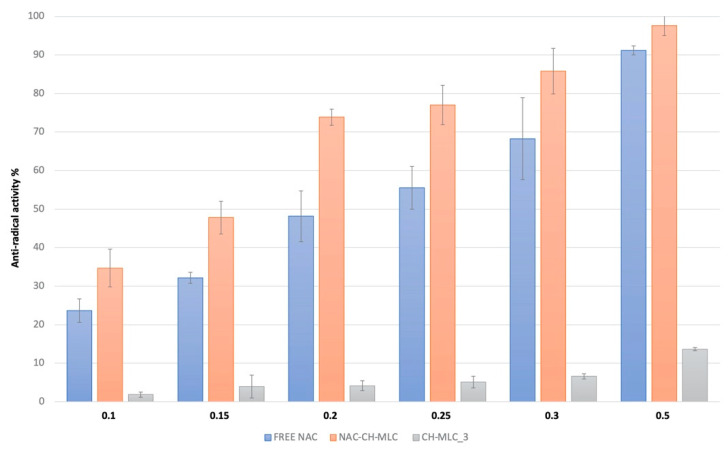
Antiradical activity percentages of free NAC, NAC-CH-MLC and CH-MLC_3 at active concentration range between 0.1 and 0.5 mg/mL.

Figure 8 describes the antioxidant activity of NAC-CH-MLC at different NAC concentrations compared with CH-MLC and NAC-free molecule activity. As expected from the EC50 results, the action of NAC inside microparticles was greater. It is possible to notice from the antioxidant activity of CH-MLC that the contribution of ascorbic acid was minimal.

### 2.6. Evaluation of the Effectiveness of MLC in the Prevention of Microbial Biofilm Formation

To exclude a toxic effect of the microparticles on bacterial cells, a microbicidal effect (ME) assay was performed using microparticles without NAC. Increasing concentrations of solid lyophilized were tested (Table 3).

By increasing the concentration of freeze-dried solid, it is noted that ME began to have a significant, albeit low, value (ME = 0.5) at 15 mg/mL.

**Figure 9:**
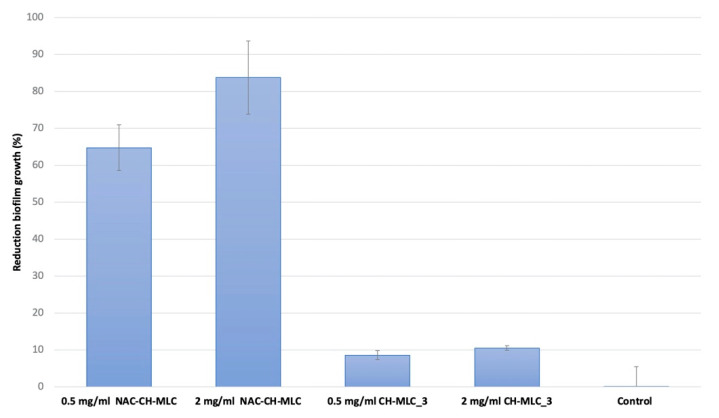
Efficacy of MLC in bacterial cell reduction. Sample concentrations are expressed as percentage of active concentration of 0.5 mg/mL of NAC-CH-MLC, 2 mg/mL of NAC-CH-MLC, 0.5 mg/mL of CH-MLC_3, and 2 mg/mL of CH-MLC_3.

Figure 9 shows the effectiveness of NAC-CH-MLC against *P. aeruginosa* biofilm formation at two different NAC concentrations (0.5 and 2 mg/mL); CH-MLC_3 placebo at two different concentrations were used as controls. Results are expressed as percentage of bacterial cell reduction with respect to bacterial growth control carried out using a nontreated metal disk. Results highlighted that a concentration of 0.5 mg/mL of active compound on NAC-CH-MLC produced a reduction of 64.74% ± 6.2% of *P. aeruginosa* biofilm growth, while the same amount of placebo MLC (CH-MLC_3) produced a reduction of only 8.57% ± 1.2% (*p* < 0.05). Furthermore, a drug concentration of 2 mg/mL was able to reduce 83.74% ± 9.95% of biofilm, compared to a reduction of 10.53% ± 0.6% made by the same amount of CH-MLC_3 (*p* < 0.05). The number of CFU on controls was always homogenous for each experiment (3.5 × 10^6^ ± 1.9 × 10^4^) and control bacteria cell reduction was negligible, around 1% ± 5.54%.

## 3. Discussion

The main aim of the present work was the development of microstructured lipid carriers (MLC) as a drug delivery system able to maintain antioxidant activity of NAC when administered topically. To fulfill this purpose, a hybrid system composed both of a polymeric core and a lipid shell was formulated.

The high shear homogenization method revealed to be an easy scalable technique, suitable for the production of microparticles with good morphology and very narrow size distribution. The addition of a cryoprotectant was essential to reduce the aggregation of MLCs during the freeze-drying process and to obtain better redispersion of the freeze-dried product. The cryoprotectant interacts with the polar heads of the surfactants and acts as a sort of “hydration shell” [39] decreasing the water osmotic activity and crystallization, favoring the glassy state of the solid sample [40]. According to literature [41,42], trehalose has proven to be the most effective cryoprotectant in preventing aggregation during freeze-drying.

The magnitude of zeta potential gives an indication of physical MLC stability: particles having a large negative or positive zeta potential will repel each other and the dispersion can be considered stable. Furthermore, the positive value of zeta potential found on NAC-CH-MLC was a positive result indicating the presence of chitosan not only in the core of the solid particles, but also on the surface, especially after swelling. The presence of CH can enhance both mucoadhesive properties of MLC to respiratory epithelium and the capability to prolong drug residence time in the target site of application [29,30].

In order to confirm the presence of chitosan, NIR analyses into a complex mixture were performed and the results obtained were encouraging.

In vitro release test was performed to understand the capability of the delivery system to let the active compound out and to define how long it took. In the present work, this test was performed in a physiological solution (NaCl 0.9%), pH 5.5 to evaluate the bioavailability of NAC in the lung. In fact, healthy respiratory tract pH is lower than in the plasma (6.15), but in CF this parameter becomes lower (5.88) due to CFTR defect, which causes a reduction of Cl^−^ transport through the cell membrane, ending in loss of bicarbonate and decrease of pH. However, the presence of inflammatory processes increasing hydrogen ions cannot be forgotten: acidity significantly rises in patients with respiratory exacerbation syndrome (5.32) [43] and lower pH increases bacterial adhesion [44].

One more important feature of microparticles applied topically in the respiratory tract is to be nonocclusive. Considering literature, solid lipid nanoparticles can exert an occlusive effect [45], and occlusive properties depend on sample volume, lipid content and particle size. In this study, results showed in Figure 8 demonstrate clearly that MLCs had no occlusive effect, showing a negative occlusion factor. The presence of CH inside the MLC, as a very hydrophilic polymer, probably permitted free water flux through the powder, avoiding occlusion.

Several studies confirmed the in vitro [46,47] and in vivo [48] efficacy of NAC that can react with the most typical radical and nonradical oxidants. The activity of NAC as an antioxidant, as well as of all other antioxidant molecules, depends on the reaction rate, which should be higher than the reaction rate of endogenous molecules and much higher than that of the other substrates. The main oxidant species involved are H_2_O_2_, ONOOH, NO_2_, HO (X), HO• and O_2_•−. The reaction rate of endogenous thiol compounds is due to the thiolate anion, which is regulated by the acidity (K_a_) of the thiol. According to this concept, NAC (pKa 9.52), compared to GSH (pKa 8.83) and cysteine (pKa 8.30), is the weakest antioxidant.

Hypochlorous acid and its derivates (HOX: hypobromous acid, HOBr, hypothiocyanous acid, HOSCN) play an important role in the pathophysiology of some lung diseases, such as cystic fibrosis (CF) [49,50]. HOX is produced by activated neutrophils and monocytes through the activation of myeloperoxidase (MPO), which catalyzes the reaction between hydrogen peroxide and halides [51]. These oxidants are bactericidal and disinfectant, helping the human response to pathogens, but they may react also with important biological molecules, inducing a cytotoxic effect. High levels of MPO protein and consequently, the increase of halogenated proteins and disulfide bonds, is reported in airway mucus of CF patients, suggesting that the oxidation occurring from airway inflammation contributes to viscous and pathologic mucus formation in affected lungs.

Hence, high concentration of NAC in this pathologic condition which sees a depletion of -SH pool, can neutralize HOX species.

NAC indirect antioxidant activity is due to the capability of the molecule to enter the cell and form glutathione (GSH). GSH is normally involved in numerous detoxifying reactions, in which it works as a substrate or cofactor of detoxifying cellular enzymes like glutathione reductase, glutaredoxin, glutathione peroxidase, peroxiredoxin, glyoxalases 1 and 2, glutathione transferase and membrane-associated proteins in eicosanoid and glutathione metabolism (MAPEG) [52]. Decrease of GSH is related to aging as well as to a wide range of pathologies, especially in the case of prolonged and severe oxidative stress and neurodegenerative disorders [53].

MLC demonstrated the ability to maintain and increase antioxidant activity of the loaded NAC. The action of NAC inside microparticles was greater than that of the free drug at every concentration tested.

Bacterial biofilms are relevant in different types of diseases, for instance, an infection of pathogens organized in biofilm, such as *A. actinomycetemcomitans* or *P. gingivalis*, has an important role in the pathogenesis of periodontal diseases; in fact, periodontitis implies an augmented immune response with the release at the local and systemic level of several inflammatory mediators which may represent an additional risk factor for the development of endothelial dysfunctions, destruction of periodontal tissue and cardiovascular diseases [11,12]. Likewise, bacterial biofilm of *P. aeruginosa* has an important role in different types of infections including chronic lung diseases like CF; once this bacillus is established in the airways, it is almost impossible to eradicate, causing considerable damage to the respiratory system [54].

In previous research, the effectiveness of free NAC against biofilm growth was already verified [54].

In the present work the influence of a microstructured carrier system on NAC capability to inhibit biofilm growth was investigated. The results were strictly correlated to the presence of CH, which slowly swells and interacts with bacteria. In fact, from literature it is well known that chitosan has antibacterial properties [55]. This action is also favored by the positive charge of the microparticles, as demonstrated from zeta potential results, which allows better interaction with the negative charge of the cell wall of the bacteria present in the biofilm matrix. Specifically, as the environment’s pH drops below the pKa of CH, electrostatic interactions between the polycationic structure of CH and the anionic component of the bacterial outer membrane are involved [56,57].

## 4. Materials and Methods

### 4.1. Materials

*Pseudomonas aeruginosa* (ATCC 17334), Tryptone Soya Broth (TSB) and Tryptone Soya Agar (TSA) from Oxoid, Basingstoke, UK; N-acetylcysteine (Farmalabor, Assago, Milano, Italy); caprylic/capric tryglicerides (Labrafac cc, Gattefossé sas, Milano, Italy) and glycerol dibehenate (Compritol 888 pastilles, Gattefossé sas, Milano, Italy); Kolliphor *p* 407, a mix of polyoxyethylene (73%) and polyoxypropylene (POE-POP) with gelling and thickening action was obtained from BASF (Germany) and polyglicery l-4 sorbitan olivate phosphate (Caldic, Milano, Italy); acetonitril HPLC grade, hydrochloric acid and orto-phosphoric acid (Sigma-Aldrich, Milano, Italy), chitosan, low molecular weight (Sigma-Aldrich, Milano, Italy), trehalose (Sigma-Aldrich, Milano, Italy).

### 4.2. Micro-Structured Lipid Carriers (MLC) Preparation

Microstructured lipid carriers were prepared by a high shear homogenization method, as reported in literature, suitably modified [58].

The experimental procedure for MLC preparation involved the preparation of 3 distinct phases, in order to obtain at the beginning a water/oil/water emulsion. The inner phase contained the active compound, N-acetylcysteine in a chitosan acidic solution.

The oily phase was prepared using caprylic/capric triglycerides as liquid lipid and glycerol dibehenate as solid lipid. They were melted in a water bath to reach a temperature of 90 °C. The aqueous phase containing POE-POP and polygliceryl-4 sorbitan olivate phosphate as surfactants was prepared and heated in order to reach the same temperature as the oil phase.

Then, the inner phase was vigorously dripped into the oily phase, emulsifying with UltraTurrax, (IKA, Staufen, Germany) at 13.000 rpm in order to form a water-in-oil emulsion. The latter was subsequently emulsified to the outer aqueous phase, always at rpm for obtaining a double emulsion. While maintaining the agitation, the emulsion was finally moved to a cold bath to obtain the solidification of the lipid matrix and the development of the MLC suspension.

#### Microparticle Purification and Freeze-Drying

MLC were purified by a gradient centrifugation method using a sucrose solution. The centrifugation process was carried out in a centrifuge model SL8R (Thermo Fisher Scientific, Monza, Italy) equipped with a fixed angle microtube rotor model MicroClick 18 × 5, at 14,000 rpm, 8 °C for 40 min. At the end of the process, the microparticles on the top of the tube were collected, mixed with a trehalose solution added to the final microparticle suspension as a cryoprotectant. The freeze-drying process of samples was carried out by placing the suspensions in −80 °C for 2 days and then in a Modulyo freeze-dryer (Edwards, Cinquepascal, Milano, Italy) at −40 °C, 10^−1^ mbar for 4 days [59].

Several batches of MLC were prepared using different concentrations of trehalose added to microparticles before lyophilization, in order to determine the best cryoprotectant concentration. Table 4 reports the percentage composition of all batches of microstructured lipid carriers prepared. All batches were prepared in triplicate.

**Figure 10:**
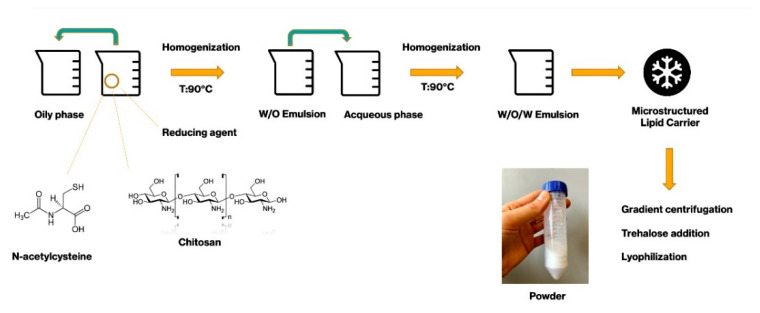
MLC preparation schematic procedure.

Figure 10 shows the schematic procedure used to obtain microstructured lipid carrier powder.

### 4.3. High-Performance Liquid Chromatography (HPLC) Analyses

To quantify the NAC amount inside the microparticles, HPLC methods from literature were used, suitably modified [35,36]. HPLC apparatus model 1100 (Agilent, Santa Clara, CA, USA) equipped with UV detector set at 210 nm, was used.

After several experiments, a symmetric (0.88) and sharp peak was obtained. Table 5 reports the optimized operating conditions used for NAC quantification.

#### 4.3.1. HPLC Method Validation

The analytical procedure validation was made in accordance with the ICH Q2 (R1) guidelines [60]. Specificity, linearity, accuracy, intermediate precision, limit of detection (LOD) and limit of quantification (LOQ) were evaluated as follows:

- Specificity was determined by comparing the retention chromatograms of a standard blank and the mobile phase.
- Linearity was assessed on 8 different concentrations, from 0.1 µg/mL to 50 µg/mL, on three consecutive days. Each concentration level was analyzed in triplicate.
- Accuracy was evaluated by choosing three different concentrations within the curve (40%, 60% and 120%) and for three consecutive days, analyses were performed in triplicate for each concentration. After calculating the NAC concentration, the percentage of recovery was calculated.
- Intermediate precision was tested with 3 analysis sessions (3 replicates for each of the three NAC concentration levels corresponding to 40%, 60% and 120%) in three different days. The three analysis sessions were carried out by three different operators to estimate intermediate precision. The average of the three recoveries was performed on 9 total replicas.

Standard deviation (*SD*) and the relative standard deviation (*RSD*%) were calculated with the formulas 1,2,3 here below reported:

Formula 1

$$X=\frac{\sum_{i=1}^{n}xi}{n}$$

Formula 2

$$s=SD=\sqrt{\frac{\sum_{i=1}^{n}{\left(xi-x\right)}^{2}}{n-1}}$$

Formula 3

$$RSD\%=\;\frac{s}{x}\;X\;100$$

where

*X* = medium value of 5 injections
*xi* = *i*-th injection value
*s* = standard deviation
*n* = number of the determinations

- Limit of detection (*LOD*) and limit of quantification (*LOQ*) were based on standard deviation of the response and the slope. The detection limit (*LOD*) and the quantification limit (*LOQ*) may be expressed by Formulas 4 and 5:

Formula 4

*LOD* = 3.3 *σ*/*S*

Formula 5

*LOQ* = 10 *σ*/*S*

where *σ* = the standard deviation of the response *S* = the slope of the calibration curve.

#### 4.3.2. Standard Solutions and Sample Preparation

For calibration studies, a calibration standard stock solution of NAC was prepared by accurately weighing out 0.010 g of NAC and dissolving it into 10 mL of a solution 10:90 (*w*/*w*) of acetonitrile and water milliQ pH 2.5. This stock solution was further diluted with the same solvent to prepare the calibration standard working solution (100 μg/mL) and consequently the dilutions 0.1, 0.5, 1, 5, 10, 30, 50 μg/mL of NAC in each sample.

### 4.4. Microstructured Lipid Carrier Characterization

#### 4.4.1. SEM Analyses

The morphological characterization of the microparticles was performed with a high-resolution scanning electron microscope (TESCAN, Mira 3 XMU, Brno, Czech Republic).

The disks were directly mounted on aluminum pin stubs by means of a graphite tape and coated with carbon using a Cressington 208 C (Cressington Scientific Instrument Ltd., Watford, GB) prior to observation. SEM analysis was performed operating at 20 kV.

#### 4.4.2. Dimensions and Zeta Potential

The dimensions, the polydispersity index (PdI) and the zeta potential of different suspensions of NAC-CH-MLC, NAC-MLC and CH-MLC were evaluated by Photon Correlation Spectroscopy (PCS) using the Zetasizer ^®^ Nanoseries (Malvern Instruments Ltd., Worcestershire, UK).

PCS analysis were performed at 90° detection angle. PdI is a parameter used to indicate the width of the size distribution ranging between 0 (monodispersity) and 1. All measurements were performed diluting appropriately MLC suspensions in distilled water (pH ≈ 5.5). At least three measurements were carried out for each sample.

#### 4.4.3. Near-Infrared (NIR) Analysis

Chitosan, trehalose, CH-MLC powder and their physical mixture with different concentrations of CH (85%, 10%, 30% *w*/*w*) were analyzed using NIR spectroscopy for further nondestructive characterization in order to reveal the presence of trehalose and CH in microstructured carriers. For NIR spectroscopy, samples were scanned in a range of the near-infrared region (950–1650 nm), in which each constituent has absorption properties, due to the stretching and bending vibrations in molecular bonds [61]. This technique presents some advantages with respect to other analytical techniques, for example, its ability to record the spectra of solid and liquid samples without prior manipulation. Furthermore, it is simple, rapid, and cost-effective [62].

For this analysis, a MicroNIR™ Pro Spectrometer, (JDSU, Milpitas, CA, USA) was used. Samples were placed on a nonreflective support with a fixed and constant distance (3 mm) from the acquisition window. Three replicates for each sample were always performed.

Data were evaluated using Unscrambler^®^ X software, version 10.4 (Camo software AS, Oslo, Norway). The spectra were pretreated by using first derivative with Savitzy–Golay smoothing followed by standard normal variate (SNV) preprocessing. Principal components analysis (PCA) on pretreated spectra was performed on the obtained spectra in order to evaluate the capability of the NIR measurement system to reveal the presence of chitosan in different samples.

### 4.5. Drug Loading (DL%) and Entrapment Efficiency (EE%)

To quantify the NAC amount inside the microparticles, 30 mg of lyophilized solid was immersed in a solution 10:90 (*w*/*w*) of acetonitrile and water milliQ pH 2.5, keeping everything at 37 °C in a thermostatic bath. After 45 min, the sample was filtrated using acetate cellulose 0.22 µm filters and analyzed with high-performance liquid chromatography (HPLC) analysis, as reported above. Results were expressed as the percentage of NAC amount with respect to the weight of the analyzed sample (DL%) and as the percentage of NAC recovered in comparison to the theoretical amount in the weighted sample (EE%). Each sample was analyzed in triplicate.

### 4.6. N-Acetylcysteine-Loaded MLC Release Study

The drug release profile from microstructured carriers was performed in NaCl 0.9% (pH ≈ 5.5). Thirty milligrams of lyophilized MLC were weighed and diluted in physiological solution, then placed inside the thermostat at 37 °C and analyzed at the following times: 15, 30, 45, 60 min and 24 h. The samples were prepared in triplicate and three HPLC analyses were performed for each sample.

Twenty-five milligrams of NAC powder were put in 25 mL of NaCl 0.9% and used as control for analyte dissolution process.

### 4.7. In Vitro Evaluation of Occlusive Properties of NAC-CH-MLC

The evaluation of occlusive properties of MLCs was performed using a gravimetric procedure [45]. For this experiment, glass beakers were filled with 15 g of water, covered with 4 overlapping cotton wool gauzes and sealed. For the two samples tested (NAC-CH-MLC and CH-MLC), two different concentrations were employed (12 and 50 mg/cm^2^). Each sample was tested in triplicate. Three uncovered beakers were used for control and 3 beakers spread with Vaseline were used as occlusive standard. The samples were incubated at 37 °C for 24 h. The weight of the beakers was recorded at 1, 3, 6 and 24 h.

The occlusion factor was computed as expressed in formula 6:

Formula 6

*F* = (*A* − *B*)/*A* × 100

where *A* is water loss through the uncovered glass (control), *B* is water loss through the covered samples.

### 4.8. Antioxidant Activity Evaluation

Antioxidant activity of microstructured lipid carriers was assessed by DPPH assay (2,2-Diphenyl-1-picrylhydrazyl), as reported in literature [63]. Antiradical activity was tested by measuring the decrease of the absorption at 516 nm of a DPPH solution after the addition of an antioxidant solution.

For each analysis, the absorbance of 50 µL of sample added to 1950 µL of DPPH reagent in ethanol was read.

To analyze the activity of the NAC molecule, NAC-CH-MLC and CH-MLC_3, they were pretreated with acetonitrile and lactic acid 0.1 M mixture (1:1 *v/v*). The suspension was filtered, and the clear solution was analyzed spectrophotometrically to give a direct measure to MLC antioxidant activity.

The decrease in absorbance was evaluated after 5 min, according with the reaction kinetics of NAC.

The amount of antioxidant needed to reduce the concentration by 50% of DPPH (EC50) and its reciprocal measurement of anti-free radical power (ARP), were calculated.

Antiradical activity % (ARA%) was also calculated through the formula 7:

Formula 7.

$$\mathrm{Antiradical}\;\mathrm{activity}\%=\frac{\mathrm{µg}\;\mathrm{DPPH}\;\mathrm{used}}{\left(\mathrm{µg}\;\mathrm{DPPH}\;\mathrm{remaining}\;+\;\mathrm{µg}\;\mathrm{DPPH}\;\mathrm{used}\right)}$$

### 4.9. Evaluation of the Effectiveness of MLC in the Prevention of Microbial Biofilm Formation

Anti-biofilm activity was evaluated on a biofilm formation step using a method already established [54]. Briefly, a suspension of *P. aeruginosa* was prepared overnight and standardized in order to have a concentration of 1 × 10^8^ CFU/mL, three dilutions (1:10) in fresh TSB were prepared from this suspension, in order to obtain 10 mL of bacterial suspension for each and incubated for three hours. Then, the bacterial suspensions were placed inside three flasks with a stainless-steel disc inside. After 24 h of incubation at 37 °C, different weights of NAC-CH-MLC and CH-MLC_3 were put in 1 mL of TSB in order to dissolve the active compound into the bacterial suspension and to have different concentrations of NAC (0.5 and 2 mg/mL) considering the final volume of 10 mL into the flasks. The prepared solutions were put into the flasks.

A suspension without any active compound was used as control. After 24 h, the suspension was removed from the incubator. The stainless steel disks were removed and placed in 10 mL of sterile water, then left in agitation for 10 min at 100 rpm. The disks were then washed with 10 mL of sterile water and placed in another 10 mL of sterile water, in which a scalpel was used for scraping the disks in order to mechanically detach the remaining adherent cells. Then, 5 min sonication in a 35 kHz ultrasonic bath was done. The suspension was diluted (1:100, 1:10,000; 1:1,000,000) and plated in TSA to count the viable cells.

The prevention of biofilm formation was evaluated by measuring bacterial cell reduction as percentage of control. The reduction percentage was calculated using the following Formula 8 [54,64]:

Formula 8

$$Bacterial\;cells\;reduction\;\%=\;100-\frac{\left(CFUc-CFUs\right)\times 100}{CFUc}$$

where *CFUc* are the colony-forming units resulting from the sum of the colonies after manual scraping and sonication of the control disk; while *CFUs* are the colony-forming units resulting from the sum of the colonies after manual scraping and sonication of the disk treated with the active compound.

Furthermore, the microbicidal effect (ME) was investigated using microparticles without NAC against a suspension of *P. aeruginosa*. *ME* was calculated using the Formula 9 [54]:

Formula 9

*Microbicidal Effect (ME)* = *log NC* − *log NE*

where *NC* is the number of *CFUs* in the control microbial suspension and *NE* is the number of *CFUs* in the microbial suspension counted after exposure to microparticles without NAC (CFU = colony-forming unit).

### 4.10. Statistical Analyses

One-way analyses of variance (ANOVA) were performed to compare multiple groups. All analyses were run using MS Excel, and differences were considered to be significant at a level of *p* < 0.05.

## 5. Conclusions

This work aimed to develop hybrid microparticles containing both a polymeric core and a lipid shell, with the aim of exerting topical action on the formation of the *Pseudomonas aeruginosa* biofilm in the respiratory tract and, in particular, in the lungs.

In previous research, the effectiveness of free NAC was verified [54]; in this work, the idea was to develop a drug-sustained delivery system with mucoadhesive properties.

The results obtained in this work permit to conclude that NAC-loaded microstructured carriers provided ideal features in order to be administered topically in the respiratory tract: small dimensions, but not so small to go directly only in the alveolar tract; positive superficial zeta potential, no occlusive effect and good antioxidant properties. Furthermore, results highlighted very high anti-biofilm activity. The effectiveness of the microparticles was greater as the concentration of loaded NAC rose. This action was also favored by the positive charge of the microparticles that allowed better interaction with the negative charge of the cell wall of the bacteria present in the biofilm matrix.

Moreover, the effective antioxidant action of the NAC-CH-MLC could allow to fight oxidative stress and therefore the appearance of the mucoid phenotype of *P. aeruginosa* and proved to be greater than that of the NAC free molecule at every concentration tested.

In conclusion, this work represents a favorable technological strategy against diseases in which bacterial biofilm is relevant, such as cystic fibrosis.

## Figures and Tables

**Table 1:** EC50 and anti-free radical power (ARP) of free NAC and NAC-loaded MLC.

SAMPLE	EC50 (mg/mL of Active Compound)	ARP (1/EC50)
Free NAC	0.23 ± 0.02	5.26
NAC-CH-MLC	0.15 ± 0.01	8.33

**Table 2:** Linear range investigated over three days.

**Day 1**			
**Replicate**	**Slope**	**y-Intercept**	**R^2^**
1	38.142	50.737	0.9969
2	34.46	53.886	0.9993
3	35.906	79.676	0.9948
**Day 2**			
**Replicate**	**Slope**	**y-Intercept**	**R^2^**
1	35.498	78.026	0.9995
2	33.805	100.07	0.9981
3	34.453	82.99	0.9997
**Day 3**			
**Replicate**	**Slope**	**y-Intercept**	**R^2^**
1	42.674	64.567	0.9998
2	42.271	73.4	0.9995
3	41.948	64.557	0.9995

**Table 3:** Microbicidal effect (ME) of increasing concentration of CH-MLC_3.

CH-MLC_3 Concentration	Microbicidal Effect (ME)
1 mg/mL	−0.0280
3 mg/mL	−0.0543
7 mg/mL	−0.0545
15 mg/mL	0.5740
30 mg/mL	0.5640
60 mg/mL	0.5300

**Table 4:** Percentage composition (*p*/*p*) of the microstructured lipid carrier (MLC) suspension (for all batches, the following substances were kept at the same concentration: POE-POP: 1.2%, glycerol dibehenate 3.2%, caprylic/capric triglycerides 1.2%, polygliceryl-4 sorbitan olivate phosphate 0.4%).

Batch	NAC (%)	Chitosan (%)	Trehalose (%)
CH-MLC_1	-	0.07	1.5
CH-MLC_2	-	0.07	3
CH-MLC_3	-	0.07	4
MLC	-	-	4
NAC-CH-MLC	1.23	0.07	4

**Table 5:** Optimized operating conditions for N-acetylcysteine (NAC) quantification in high-performance liquid chromatography (HPLC).

Parameter	Conditions
Chromatographic column	Waters spherisorb^®^ 5 µm ODS1 4.6 × 150 mm
Flow rate	0.8 mL/min
Column Temperature	25 °C
Injection volume	50 µL
Wavelength	210 nm
Run Time	5 min
Mobile Phase	Aqueous phase prepared adjusting the pH to 2.50 with orthophosphoric acid and organic phase (acetonitrile) in a volume ratio of 90:10.

## Data Availability

The data presented in this study are available on request from the corresponding author. The data are not publicly available due to PhD project still in progress.
